# Legal Notes for Institutional Workers

**Published:** 1912-05-25

**Authors:** 


### LEGAL NOTES FOR INSTITUTIONAL WORKERS.

(The Editor will be pleased to answer Legal queries in this column.)

### A Post where Qualifications are Superfluous.

The fact that the office of Coroner for Westminster has been filled by one who has the double qualification of a medical man and of a barrister may still lead people to suppose that such, or in any case some, qualifications are necessary in order to fill the important post of "King's Crowner." But no qualification is necessary. The "youngest son of a youngest son" might legally be jobbed into the billet, even if he had failed at every examination he ever sat for, and he could be chosen for life or during good behaviour. In their report presented in 1908, a Departmental Committee of the Home Office made a suggestion that no one should be appointed a coroner who is not a barrister, solicitor, or medical man; but this report is still in its pigeon-hole. When we look back through the dim corridors of history, the reason why a coroner need formerly have possessed no particular qualifications becomes apparent. In the year 1194 the real office of the coroner was not so much to administer justice as to guard what may be called chance revenues falling to the King, such as forfeited chattels of felons, deodands, wrecks, royal fish, and treasure trove; while the inquisition in cases of persons dying suddenly was held chiefly to learn whether any property escheated to the Crown. Having regard to the duties of the early coroner, any hanger-on at Court would have done the King's work as well as, or even better than, any doctor or lawyer! But he had other peculiar duties. Thus, according to the "Mirror of Justices": "Coroners were wont also to hold their views in cases of sodomy, and on infant monsters who had nothing of humanity, or who had more of the beast than the man in them; and these the coroner caused to have buried. But the holy faith grows stronger every day, whereby folk do not burden their souls with such horrible sins so commonly as they used." Owing to the fact that the County Coroner is now elected by the County Council on a writ "de coronatore eligendo," the chance of jobbery in connection with the office is much less real in practice than it is possible in theory.

### Medical Protection Societies and the Law.

The case of *Square* v. *London and Counties Medical Protection Society* (*Ltd.*), which reached the Court of Appeal two weeks ago involved a question of considerable interest to members of medical protection societies. The material facts may be very briefly stated. The plaintiff, Dr. W. H. Square, is himself defendant in an action for libel which is now pending against him at the suit of Dr. R. S. Pearson. As Dr. Pearson is a member of the London and Counties Medical Protection Society that body undertook the conduct of the action upon his behalf in accordance with the terms of their memorandum and articles. The present action was commenced by Dr. Square, in which he asked for an injunction to restrain the defendant company, its servants or agents from unlawfully "maintaining" Dr. Pearson in his action. The judge at chambers having granted an injunction, the Court of Appeal discharged it on the ground that upon the facts disclosed there was only a *primâ facie* inference of maintenance. Lord Justice Vaughan Williams said that when the articles of association were looked at he thought it possible and very likely that no offence of maintenance had been committed. As this does not finally dispose of the matter we refrain from comment.

### Legal Meaning of Maintenance.

The following definition of "maintenance," however, is interesting: It has been defined as an offence which consists in officiously intermeddling in a suit that in no way belongs to one, as by maintaining or assisting either party with money or otherwise, although having nothing to do with it. For maintenance a person may be prosecuted, and an action may be brought against him for damages caused by his act of maintenance. There are, however, many exceptions to maintenance, and it has been held that a rich man may out of charity assist a poor man to maintain a right which he would otherwise lose.

